# Supplementary material for: Regulatory element modules as universal features for single-cell chromatin analysis
Source: bioRxiv. 2025 Dec 12:2025.12.10.692786. Preprint. [Version 1] doi: 10.64898/2025.12.10.692786 (PMC12710825; doi:10.64898/2025.12.10.692786)
Supplement: 9 [file NIHPP2025.12.10.692786v1-supplement-9.pdf]

bioRxiv preprint doi: https://doi.org/10.64898/2025.12.10.692786; this version posted December 12, 2025. The copyright holder for this preprint (which was not certified by peer review) is the author/funder, who has granted bioRxiv a license to display the preprint in perpetuity. It is made available under aCC-BY 4.0 International license.

Supplementary Figures

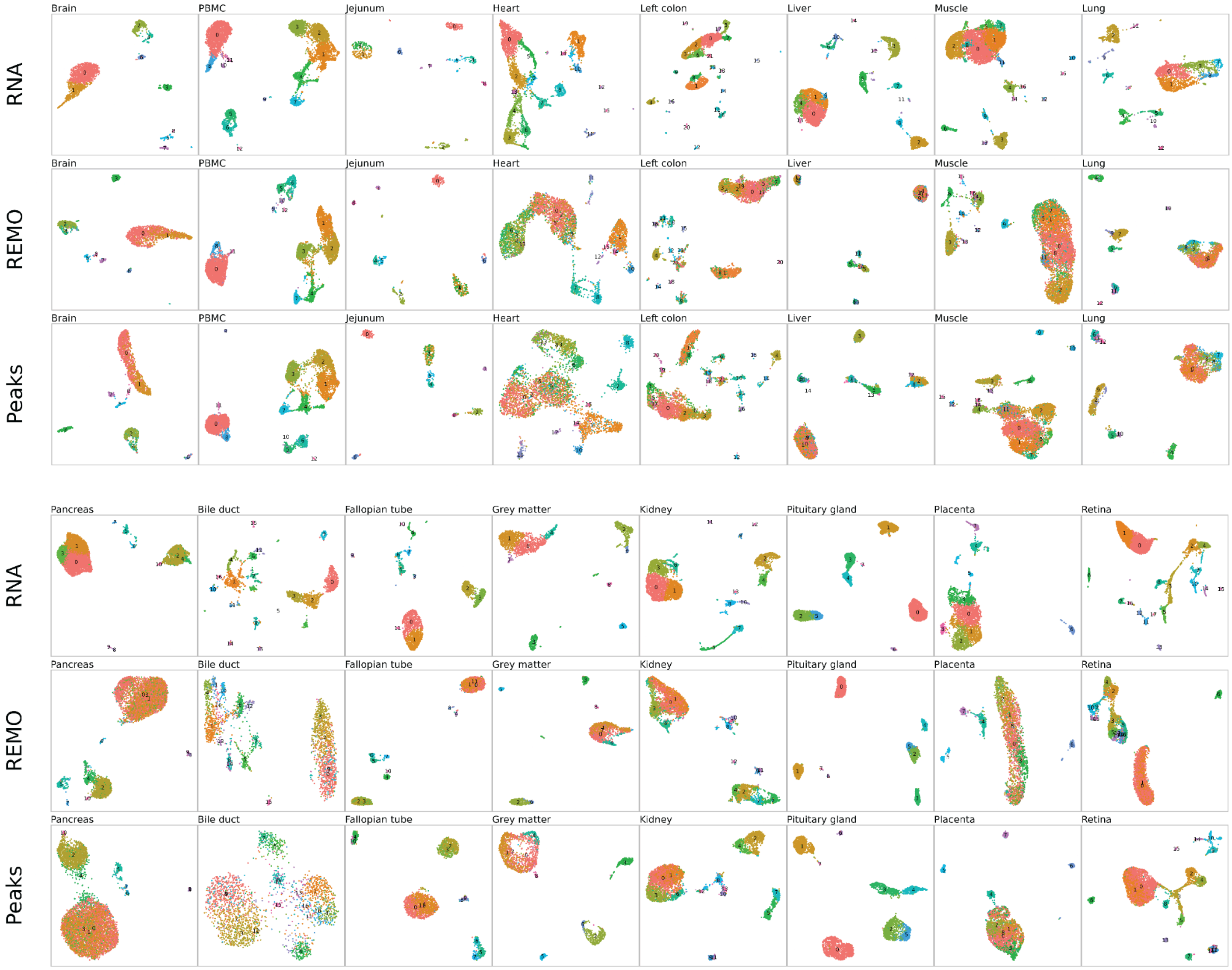

Supplementary Figure 1: UMAP representations for all multiome datasets

Two-dimensional UMAP representation for 16 multiome datasets processed using gene expression data (RNA) or DNA accessibility data either using REMO or peak calling. For each dataset, cells were annotated by gene expression-based clustering.
